# Supplementary material for: Older people’s perceptions of the impact of Dance for Health sessions in an acute hospital setting: a qualitative study
Source: BMJ Open. 2021 Mar 26;11(3):e044027. doi: 10.1136/bmjopen-2020-044027 (PMC8006823; doi:10.1136/bmjopen-2020-044027)
Supplement: Supplementary data [file bmjopen-2020-044027supp001.pdf]

Interview Guide Participants

1. How many of the dance (music and movement) sessions that take place on the ward have you joined?
2. Who told you about the group and invited you to join?
3. Do you ever dance when you are at home?
4. Have you had previous experience of dancing? *(Can you tell me what dancing you did and when?)*
5. If you were telling your next door neighbour about the dance (music and movement) sessions on the ward what would you say about them?
6. What are the best bits about the dance (music and movement) sessions?
7. Are there any changes you would like to see? What would make the sessions better?
8. Some people have commented on the following; what do you think about:

   The time/venue/frequency of the sessions?

   How long the session is?

   The music used for the sessions? *(volume? repertoire?)*

   The clarity of explanations? *(How clear is it as to what is happening?)*

9. Some of the participants have mentioned that the sessions affect them in a positive way others find them quite demanding what do you think?

   For example:

   Have you got to know the other patients better?

   Have you got to know the staff better?

   Enjoyed moving to the music?

   Bringing back memories?

   Learning new things?

   Any physical effects (sleep/appetite/fatigue)

10. Is there anything else you would like to say about the dance (music and movement) sessions?
