# Supplementary material for: Older people’s perceptions of the impact of Dance for Health sessions in an acute hospital setting: a qualitative study
Source: BMJ Open. 2021 Mar 26;11(3):e044027. doi: 10.1136/bmjopen-2020-044027 (PMC8006823; doi:10.1136/bmjopen-2020-044027)

Supplementary File

Description of the Dance for Health Programme

The Dance for Health for older people programme takes place on wards in the Department of Elderly Medicine ward, the Diabetes and Endocrine Medicine ward, and the Stroke and Rehabilitation Unit in a large acute hospital Trust. The dance sessions are weekly and scheduled for an hour on each ward and usually take place in the ward day rooms. The number attending the sessions ranges between 4-12 patients and is dependent on the available space and staff support. The sessions are introduced to patients by the ward staff as music and movement. There is no exercise regime imposed and no choreography. Patients are invited to participate in the activity to the degree that they feel comfortable and choose whether to attend the sessions or not.

The dance sessions aim to improve the hospital experience of older people and to enhance their wellbeing and physical health. They are delivered by a Dance Artist who is a trained Dance Movement Therapist, but the sessions are not offered as a psychotherapeutic intervention. The Project Coordinator, who is also the Falls Prevention Co-ordinator for the Trust, supports the sessions through both liaising with wards where the sessions take place and by helping wards to identify patients who may benefit from taking part in the sessions. The sessions are open to all inpatients on the wards where the programme takes place, including people with Parkinson's disease, delirium, unstable diabetes, stroke, falls and dementia. The Dance Artist and Project Coordinator provided information and training for ward staff to raise awareness of the programme and engage staff in the sessions.

The dance activity takes place in a circle formation so participants are close to the person next to them and can see others in group. The sessions always start with the Dance Artist introducing the session and stating the day of the week and the date, followed by the location of the activity. She then asks all those present to introduce themselves in turn. The first music track played is an instrumental piece and the participants are encouraged to start some gentle movements to warm up their hands, arms, and shoulders. During the following piece of music, lower body movement is encouraged. Participants are then invited to choose a music track; this means that some of the music is familiar to those taking part. The process of choosing music also helps to stimulate conversation between the participants. The middle of the session is livelier, using music with a more upbeat tempo. This encourages more movement and participants may also

join in singing to some of the tracks. Towards the end of the session, slower quieter music is used during the cool down phase and to help relax the participants. During this relaxation time, patients are given a gentle massage to their neck and shoulders by the staff present, and participants are invited to close their eyes and listen to the quiet, gentle, instrumental music played at this time.

Table 1: Example of the coding process and theme development

Data Extract	Code	Sub-Theme	Theme
"You could think with the music and you could develop your own system of stretches, it sort of guided you into the movements you see. Yes, the music guided you into different things to think of the areas that needed stretching, you see, the bits that hurt. Some of the music gave you the impetus to move and stretch………You are doing like you are in the water, like you are swimming, that you're in freestyle".	Moving and stretching<br>Inspired to move	Encourages Movement	Physical Impact

Figure 1: Thematic Map

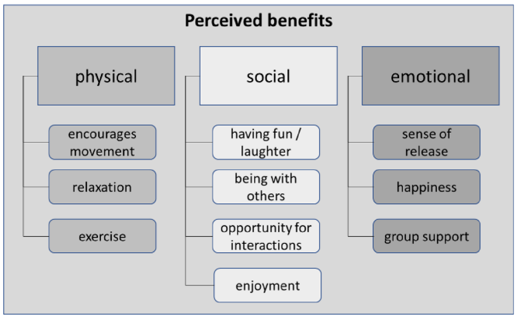

Supplement: Supplementary data [file bmjopen-2020-044027supp002.pdf]
