# Supplementary material for: Older people’s perceptions of the impact of Dance for Health sessions in an acute hospital setting: a qualitative study
Source: BMJ Open. 2021 Mar 26;11(3):e044027. doi: 10.1136/bmjopen-2020-044027 (PMC8006823; doi:10.1136/bmjopen-2020-044027)
Supplement: Author's manuscript [file bmjopen-2020-044027.draft_revisions.pdf]

BMJ Open

BMJ Open is committed to open peer review. As part of this commitment we make the peer review history of every article we publish publicly available.

When an article is published we post the peer reviewers' comments and the authors' responses online. We also post the versions of the paper that were used during peer review. These are the versions that the peer review comments apply to.

The versions of the paper that follow are the versions that were submitted during the peer review process. They are not the versions of record or the final published versions. They should not be cited or distributed as the published version of this manuscript.

BMJ Open is an open access journal and the full, final, typeset and author-corrected version of record of the manuscript is available on our site with no access controls, subscription charges or pay-per-view fees (http://bmjopen.bmj.com).

If you have any questions on BMJ Open's open peer review process please email info.bmjopen@bmj.com

BMJ Open

Older people and dance in an acute hospital setting: A qualitative study

Journal:	*BMJ Open*
Manuscript ID	bmjopen-2020-044027
Article Type:	Original research
Date Submitted by the Author:	25-Aug-2020
Complete List of Authors:	Bungay, Hilary; Anglia Ruskin University, Allied and Public Health<br>Hughes, Suzanne; Anglia Ruskin University, Allied and Public Health
Keywords:	GERIATRIC MEDICINE, QUALITATIVE RESEARCH, REHABILITATION MEDICINE

SCHOLARONE™
Manuscripts

I, the Submitting Author has the right to grant and does grant on behalf of all authors of the Work (as defined in the below author licence), an exclusive licence and/or a non-exclusive licence for contributions from authors who are: i) UK Crown employees; ii) where BMJ has agreed a CC-BY licence shall apply, and/or iii) in accordance with the terms applicable for US Federal Government officers or employees acting as part of their official duties; on a worldwide, perpetual, irrevocable, royalty-free basis to BMJ Publishing Group Ltd ("BMJ") its licensees and where the relevant Journal is co-owned by BMJ to the co-owners of the Journal, to publish the Work in this journal and any other BMJ products and to exploit all rights, as set out in our licence.

The Submitting Author accepts and understands that any supply made under these terms is made by BMJ to the Submitting Author unless you are acting as an employee on behalf of your employer or a postgraduate student of an affiliated institution which is paying any applicable article publishing charge ("APC") for Open Access articles. Where the Submitting Author wishes to make the Work available on an Open Access basis (and intends to pay the relevant APC), the terms of reuse of such Open Access shall be governed by a Creative Commons licence – details of these licences and which Creative Commons licence will apply to this Work are set out in our licence referred to above.

Other than as permitted in any relevant BMJ Author's Self Archiving Policies, I confirm this Work has not been accepted for publication elsewhere, is not being considered for publication elsewhere and does not duplicate material already published. I confirm all authors consent to publication of this Work and authorise the granting of this licence.

For peer review only

Older people and dance in an acute hospital setting: A qualitative study

Authors: Hilary Bungay[1]* and Suzanne Hughes[1]

Affiliations: School of Allied and Public Health, Anglia Ruskin University Cambridge

*Corresponding author

Author Email Addresses

- Bungay Hilary: hilary.bungay@anglia.ac.uk
- Hughes Suzanne: suzanne.hughes@anglia.ac.uk

Key Words: Dance, older people, music, movement, hospital, acute care, physical activity, social interaction, emotional support.

Manuscript word count: 3694

For peer review only - http://bmjopen.bmj.com/site/about/guidelines.xhtml

Original Protocol/topic guide

See attached (supplementary file)

Funding Statement

The Dance for Health Programme was funded by the Dunhill Medical Trust (N187/1116) and included funding for evaluation. The study was commissioned by the Head of Arts via the Addenbrookes Charitable Trust, and the programme delivery team were involved in the study design, but had no input into the data collection, management, analysis or interpretation of the data, review or approvals of the manuscript; or decision to submit the manuscript for publication.

Competing interests

No conflicts of interest declared.

Author Contributions

HB led the study including the design, ethical approval, and observed dance sessions and conducted interviews. SH observed sessions and conducted interviews and contributed to the development of the coding framework. Both HB and SH conducted data analysis and data interpretation. HB wrote the first draft of the manuscript and SH reviewed and suggested improvements to the manuscript draft. Both authors contributed to reviewing and editing the manuscript and approved the final manuscript for submission.

Reporting Checklist

See attached COREQ checklist

For peer review only - http://bmjopen.bmj.com/site/about/guidelines.xhtml

Data Sharing Statement

Data from this study are not being made available for sharing as participants were not asked for specific consent for this as part of the consent process

Patient and Public Involvement

Patients and their relatives or other members of the public were not involved in the design of the study; however, the study was focused on patients and their relatives' perceptions of the programme.

For peer review only

For peer review only - http://bmjopen.bmj.com/site/about/guidelines.xhtml

Abstract

Objectives: To explore the perceptions of hospitalised older people and their relatives of the impact of taking part in group dance sessions in an acute hospital setting.

Design: A qualitative descriptive approach was used.

Setting: An acute hospital Trust in the East of England.

Participants: Purposive sample of dance group participants

Intervention: Weekly music and movement sessions for frail older people on Department of Elderly Medicine Wards and the Stroke Rehabilitation Unit. Sessions take place in the ward and are facilitated by a dance artist supported by ward staff.

Results: Twenty-one semi-structured interviews were conducted with older people and/or their relatives. Thematic analysis of the data identified three overarching themes: (1) Dance as a physical activity, (2) Dance as an opportunity for social interaction and (3) the dance group as a source of emotional support.

Conclusion: Participants perceived that dance provided physical, social, and emotional impacts. Sessions were valued as an enjoyable way to undertake physical activity and provided an opportunity for social interaction between patients. This is important as loneliness and boredom are a common occurrence when hospitalised and are detrimental to overall health and wellbeing. Patients reported an emotional impact though taking part; happiness from engaging with the group, and the release of pent up emotions through the triggering of memories by the music and conversations within the group. Further research is required to investigate the effectiveness of group dance sessions in increasing physical activity on an acute ward, and the potential psychological benefits for hospitalised older people.

For peer review only - http://bmjopen.bmj.com/site/about/guidelines.xhtml

Article Summary: Strengths and Limitations of this study

- The first study of the impact of attending a dance group on older people in an acute hospital setting.
- An exploration of in-patient experience using semi-structured interviews.
- The study provides a rationale for investigating how dance in hospital could be used to increase levels of physical activity and to help alleviate patient boredom on acute hospital wards.
- The study took place in one large teaching hospital in the East of England, this may limit transferability to other institutions and cultures.

For peer review only

For peer review only - http://bmjopen.bmj.com/site/about/guidelines.xhtml

Introduction

When older people are hospitalised, they experience a loss of autonomy, their quality of life can be adversely affected, and they may experience functional decline due to their ability to move around being restricted or limited due to ill health. During hospitalisation older adults may spend 83% of their hospital stay in bed [1] and this lack of mobilisation may result in 'deconditioning syndrome' [2]. The hospital environment discourages physical activity with much of the care taking place at the hospital bedside [3]. In the United Kingdom recognition of this has led to recent initiatives such as "End PJ Paralysis" (where PJ stands for Pyjamas), with patients being encouraged to get out of bed and into their clothes to reduce excessive bed rest [4]. The restricted opportunity for movement can also mean that there is little opportunity for engagement in social interaction. This can lead to boredom, a common occurrence in hospitalised older adults, resulting in low mood [5] and cognitive decline [6].

Dance is a physical activity which not only employs motor skills, but also engages cognitive functions, including perception, emotions, and memory [7]. Research on dance with older people, has been conducted in a range of settings and across the globe [8-14]. But there are a limited number of studies that include frail or functionally impaired adults [15]. Furthermore, not all studies have identified positive outcomes, and there are methodological weaknesses in some of this research with small sample sizes, lack of transparency in the reporting of qualitative data analysis, and heterogeneity of tools and scales used.

A large hospital Trust in the East of England introduced a weekly *Dance for Health* programme. The underpinning philosophy for *Dance for Health* is that it is about dance and enabling people to be creative and expressive rather than being just an exercise programme or a therapeutic intervention. The sessions are facilitated by a dance artist and take place weekly on wards in the Department of Elderly Medicine and the Stroke and Rehabilitation Unit. They

For peer review only - http://bmjopen.bmj.com/site/about/guidelines.xhtml

are introduced to patients as Music and Movement sessions. There is no exercise regime imposed and no choreography, but patients are invited to move and express themselves, and to participate in the activity to the degree that they feel comfortable. Further details of the sessions are presented elsewhere [16, 17].

Method

Research Design

A qualitative exploratory descriptive approach within an interpretive framework using semi-structured interviews was used to explore patients' perspectives and experiences of participating in *Dance for Health.* The study data presented here formed part of a larger service evaluation which adopted a mixed methods approach, including structured observation and interviews with health care professionals [16, 17].

Hospital staff invited patients and relatives who had attended the dance sessions and were cognitively able to take part in the interviews to speak to the evaluation team. Participants received a participant information sheet and provided written consent to be interviewed. As an evaluation involving an intervention in current use meeting the Health Research Authority criteria of an evaluation [18], the project was registered with the Safety and Quality Support Department at the Cambridge University Hospitals Trust (PRN:6594). It was also approved by the ARU University Faculty Research Ethics Panel (FREP).

Data Collection

To answer the specific objectives of the study participants were asked about their likes/dislikes about the sessions and the effects they felt taking part had on their overall wellbeing and physical health. The interviews were face to face and the length ranged from 5-33 minutes. Most interviews took place following dance sessions, except four which took place the

For peer review only - http://bmjopen.bmj.com/site/about/guidelines.xhtml

following day. Interviews were conducted in the day room where the activity had taken place or in the ward bay. The interviews were semi-structured, and audio-recorded and transcribed verbatim for analysis. Transcripts were anonymised using pseudonyms and checked for accuracy against the original recording.

Data Analysis

Data analysis process followed an interpretive approach, and to answer the specific objectives of the study started with pre-defined aims and objectives. The data was also explored to ensure the analysis reflected the accounts of the participants and responded to new themes as identified. Thematic analysis followed Clarke and Braun's six stages of analysis and was conducted manually to ensure rigour and trustworthiness [19]. To increase dependability and credibility the first three interviews were listened to by both authors for consistency before further interviews were undertaken. To further ensure dependability transcripts were checked for accuracy with the audio recordings. This also familiarised the authors with the data. To ensure trustworthiness of analysis both authors independently read each interview transcript and made notes of potential codes, and through discussion a coding framework was created. The transcripts were then subject to detailed coding by HB, and the coded data was then searched for patterns to develop sub-themes which were then clustered and organised into the final themes by both authors.

Results

Twenty-one patients and/or relatives were interviewed by either HB or SH, who had also attended sessions and undertaken structured observations. Two of those interviewed were men, and the age of those interviewed ranged from 60s to 92. Participants were not asked their age, but some volunteered this information during the interview and the ages of other participants were estimated (by decade) by the team during the interview interaction.

For peer review only - http://bmjopen.bmj.com/site/about/guidelines.xhtml

For peer review only

Three main themes were developed from the data: Dance as a physical activity, Dance as an opportunity for social interaction and the dance group as a source of emotional support. Where relevant the reported findings are supported with anecdotal observations from the sessions. The names used throughout are pseudonyms to protect the identity of the participants.

Dance as a physical activity

Patients participating in the sessions were elderly and mostly frail, with restricted mobility due to their physical health. The majority remained seated throughout the dance sessions, moving their arms and legs to varying degrees to the music. Nearly two thirds (13) of those interviewed, when asked to describe what they liked about the sessions, referred to the physical impact and the opportunity for activity the sessions provided. The dance sessions were seen to be beneficial in terms of loosening up stiff muscles and joints, with recognition that being in bed for long periods of time leads to the body becoming stiff and resistant to movement.

"Yes, everything. Yes, because it helps you to move your limbs and get you going. As I said, I've got no use- my hands lock, and you drop things sometimes. The exercise helps your hands, everything….. Yes, get your body going". (Ellen)

"It just helps everything. Helps the muscles, and the mind and everything….it just relaxes you" (Angela)

The music which accompanies the sessions encourages movement, and participants would sway in time to the music and mirror the movements of the dance artist and others in the group. Many of the participants would improvise their own moves with others copying these actions. Even patients who were very frail and unable to make extensive movements would adjust their posture and would sit up being noticeably more upright in their chairs. For some, upbeat music encouraged rhythmic movement, whilst others responded to slower tracks by stretching their limbs.

For peer review only - http://bmjopen.bmj.com/site/about/guidelines.xhtml

"You could think with the music and you could develop your own system of stretches, it sort of guided you into the movements you see. Yes, the music guided you into different things to think of the areas that needed stretching, you see, the bits that hurt. Some of the music gave you the impetus to move and stretch……..You are doing like you are in the water, like you are swimming, that you're in freestyle". (Carol).

Whilst some attributed their movements in the sessions to the music that was played, it was also seeing others in the group move which inspired them to try and move more.

"Yes, power in it, in the music. To be honest with you, it is the music that makes you want to dance, you know?" (Maureen)

"It's uplifting, and it's just rewarding, and movement was great because I fell on my right side of my body. So, just moving my arms slowly was fantastic, but I met the other patients there, not all from (ward 1), some from (ward 2). It was lovely, and I thought, you know, "I just feel horrible, and I feel I'm the only sad person in here," and you look at other people, and some people are worse. I'm really grateful. Not grateful that I saw them, but these other people are struggling as well… Looking at other people and thinking, "Well, you know what? I can do this and I can do that." (Alex)

"Wanting to do something. Even just standing up in the chair, suddenly, was a massive thing for people, but they wanted to do it, because they felt motivated, inspired, feeling a bit alive, by the whole situation". (Relative 2)

This relative also went on to suggest that the sessions were like a form of physiotherapy without participants realising that they were engaging in physiotherapy.

It was interesting that those interviewed referred to how they felt other patients benefited from taking part in the sessions. This mostly related to enjoyment, but also the benefits of the physical movement.

For peer review only - http://bmjopen.bmj.com/site/about/guidelines.xhtml

"Oh lots of ways, but it helps them to get moving and moving their limbs, and I've seen (name) lifting her legs up and things like that" (Ellen)

"Everyone was able to do the movements that you gave, and going from the sublime to a bit more jazzy it was beautiful". (Mary)

Three of those interviewed referred specifically to exercise, including Collette who called the movements' 'exercises'.

"I think, everybody joined in and were very happy, and they did their exercises, which is lovely. It was very, very nice".

Dance as an opportunity for social interaction

While the beneficial physical impact of the sessions was acknowledged by participants, the sessions were also valued for the opportunity for the social interactions the sessions provided. The dance activity takes place in a circle formation so people are sitting close to each other and can see others in the group. Often a sense of fun was apparent, as many of the interactions generated laughter within the group. Such group interactions had a positive effect on those interviewed…

"I thought it was very therapeutic…. Something up here made you feel you were with it and you felt involved. That's how I felt. I didn't feel lonely or shut out. I felt it was company" (June)

"We were laughing and talking as well, and I think that helped too. It generated a community feeling, do you know what I mean? (Margaret)

Others remarked that it had given them an opportunity to get to know other people on the ward and the two men particularly valued this.

"I found the session today, which I was a bit doubtful about to start with, very interesting. Because to start with, I learnt the names of patients who were in the same ward as me but who

For peer review only - http://bmjopen.bmj.com/site/about/guidelines.xhtml

you don't see when you're lying in bed all day….I learnt their names and I found the music had an enlivening effect on everybody…".(David)

"Even those who were perhaps more disabled than others. So I thought it was a very good session. I thought we bonded together….( ) Well, you're lying in bed and when you can't get up and walk around, you can't talk to people." (Lionel)

The impact of the session extended beyond the allotted time for the session. In one wards, three women who were taking part in the session just before lunch struck up a conversation, and decided to stay in the day room following the session to have their lunch together. One of the relatives interviewed also described how, following a session, people in the ward bay who had joined the session were talking about it during the rest of the day. She also remarked how they found common ground because of the session and the mood was buoyant as a result of joining in. Another relative who was interviewed with her mother also commented on how it was good that the patients could get together in a fun activity.

"I thought it was brilliant. And I think it's so good to get the people together as well because they interact with each other and they do things together and it's fun. I thought it was brilliant". (Relative 1)

This same relative also commented on how she liked the fact that she was able to do the session with her mother, and how it gave them something to do together, which was increasingly unusual as her mother aged.

The dance artist would ask people if they would like to hear a piece of music and this would often trigger conversations as people requested certain tracks. In addition, when people recognised certain tunes this could evoke memories that initiate further conversations and laughter in the group. Through these conversations people got to know each better and in some cases, they found that they had previously been at the same school or lived in the same road

For peer review only - http://bmjopen.bmj.com/site/about/guidelines.xhtml

many years ago. As well as providing a sense of community for individuals who experienced loneliness at home, the sessions gave people the opportunity to make new friends

"I enjoyed being with the people, different people" (Margaret)

"It's fantastic being with other people and just chat with other people" (Alex)

"Because I am registered disabled, so don't go a lot. I used to be sociable, and I have lost all my friends now, so this is really nice" (Polly)

The dance groups as a source of emotional support

The sessions were often happy and relaxed with conversations and different songs generating amusement and laughter. However, for some participants the music triggered sadness and sometimes tears, although for others the tears were brought on through happiness. One such case was observed when a patient said at the end of a song "*it brought tears to my eyes.. I love togetherness, sharing happiness"* in another example when dancing to 'Love me Tender' one patient cried and said *"but I enjoyed it … it really touched me"*. One of the men who was interviewed reflected on how the songs reminded him of his youth, making him feel sad.

"I felt a bit tearful when we played songs that took me back to my youth, really". (Lionel)

Although some patients did become sad when certain songs were played, this was not necessarily a negative impact. For example, Maud said that since she had been in the hospital she had felt that she had had a lump in her chest because she was bottling her emotions and concerns about what was wrong with her. She went on to explain that she had cried in the music and dance session, experiencing a sense of release and feeling that it was safe to cry in front of others who were going through the same thing as herself.

"It was very uplifting, and you know. Tearful for me... Everything bottles up, I think. Coming in hospital, and it sort of brings it out. And its cleansing. I feel better for it….because I kind of

For peer review only - http://bmjopen.bmj.com/site/about/guidelines.xhtml

had a lump here, that it was bottling up…I didn't know what the outcome of that was going to be. So, I just found it very, as I said, uplifting and healing. I don't know. I think lots of stuff goes on in your head when you come into hospital. When you don't know really what's wrong. ….. but it's a worrying time for the family. Yes, bottled up, and you know. But being able to talk about it. And cry, you know, even cry in silence. It's just cleansing. Being here, yes. Among other people who are going through the same, or more or less the same things, you know". (Maud)

If participants were upset the rest of the group were always very supportive and offered words of encouragement, this included touching the person's arm or taking hold of a hand. Such actions are a form of emotional support that is provided by a being part of group and can help people to cope with stressful situations.

Most participants however, appeared to really enjoy the sessions, and spoke about how much they had enjoyed the dance and found it uplifting,

"and I really enjoyed that. It really lifted up my spirits. Honestly, I love it. I've been feeling so low, and it just helped me build up, and I feel so positive now". (Alex)

"It made me feel so wonderful afterwards. I felt like I was walking on air". (Polly)

"It was very enjoyable. I found it relaxing and reviving at the same time, if you know what I mean. And thinking and coordinating and everything else which is something you don't practice". (Dora)

"...It was a real enjoyable session. I really enjoyed it. ...Bearing in mind that I've been stuck in a room in a hospital for about four or five months. This was an outlet". (David)

"I thought it was brilliant. [….] Yes, it really gave me a lift", (Gertie)

For peer review only - http://bmjopen.bmj.com/site/about/guidelines.xhtml

Again, participants also talked about the impact on others of taking part and how they had observed people coming to the sessions 'grouchy' and 'grumpy', yet through taking part their demeanour changed significantly and people appeared more cheerful and happier.

"and all the other patients were lively and bright too, what they were when they came in. They were all flat and miserable, and they livened up. They were totally different. Yes. They looked totally different when they left". (Mary)

Discussion

In this study, the dance sessions were perceived to be an activity which provided participants with an opportunity to increase their levels of physical activity. Patients enjoyed the opportunity to interact with others on the ward, and despite some sadness, the majority enjoyed the sessions and experienced a positive affect through taking part.

The perceptions of those interviewed were that the dance sessions were beneficial to their health and wellbeing. A major strength of this study is that it presents the findings from an innovative programme of music and movement for older people in an acute hospital setting. Semi-structured interviews with patients and relatives provide insight into the perceived benefits of the programme, and a rationale for exploring how such programmes could be introduced to increase levels of physical activity and to help alleviate patient boredom on acute hospital wards.

As with all research there are limitations to this study. Interviews were kept short, not only because they were designed to answer specific questions for the funder, but also because participants were frail and easily fatigued. In addition, only patients who participated in groups were interviewed and the responses were all very positive. It would have been interesting to interview those who chose not to attend the sessions and to explore their reasons, particularly as more women than men attended the sessions each week.

For peer review only - http://bmjopen.bmj.com/site/about/guidelines.xhtml

For peer review only

The patients participating in the Dance for Health groups were often very elderly (85+) and frail, and had been hospitalised for a number of weeks, and so remained seated for the activity. We could find no evidence in the literature on the effectiveness of seated dance as a physical activity for older people, although a systematic review found that dance can have multiple physical benefits for older people, including aerobic power, muscle endurance, strength and flexibility of lower body and balance [20]. Our participants expressed understanding of the need to try and maintain their physical abilities. This resonates with the work of Lafrenière et al., who found that older hospitalised adults were cognisant of the risks of functional decline utilising various strategies to maintain both physical abilities and good spirits [21]. Experiencing things that are familiar and 'normal', such as dance and listening to music, when in an uncertain and stressful environment can also help people to maintain a sense of self. Furthermore, pleasure and enjoyment, a sense of connection, are linked to mental wellbeing [22], and social engagement and improving psychological health has been found to lead to positive health outcomes [23].

It has been recommended that purposeful meaningful activities should be included in multi-component interventions designed to increase physical activity of patients during a hospital stay [3]. As outlined previously, hospitalised older people can have low levels of activity, which results in deconditioning, and can lead to longer hospital stays, and possible further loss of physical function affecting recovery post-discharge [24]. It is therefore imperative that care providers consider alternative forms of exercise such as dance to enable and encourage hospitalised older people to engage in physical activity. A group activity such as dance, which is seen as fun rather than therapy could be an attractive option for patients, particularly as the arts tend to be associated with normative pleasurable experiences making them more attractive, accessible and acceptable than conventional care [25].

For peer review only - http://bmjopen.bmj.com/site/about/guidelines.xhtml

Conclusion

This paper presents the findings from a study of an innovative programme of dance activities introduced onto Elderly Medicine wards and Stroke and Rehabilitation Unit in an acute hospital Trust. As discussed in the introduction, there exists research which demonstrates the impact of dance on the health and wellbeing of older people in the community and in residential care. The study focussed on the experiences of hospitalised older people and their relatives taking part in the dance sessions. It was not possible to conduct a controlled study to look at the impact on the number of falls, whether physical activity increased, or if the rate of rehabilitation improved because of taking part. However, what the study demonstrated is the value placed on the activity by the participants in terms of positive affect and the social opportunity the sessions afforded. In addition, and of significance, is also how the patients recognised the importance of maintaining physical function and how the dance activity encouraged them to move. Further research exploring the use of dance with hospitalised older people would be valuable to investigate whether overall physical activity levels do increase as a result of the dance activity, with measurement of both physical functioning, and the psychological wellbeing of participants undertaken.

Acknowledgements

We would like to thank the patients who were interviewed for this study, and Damian Hebron (Head of Arts), Filipa Pereira-Stubbs (Dance artist), and Deborah Quartermaine (Project Co-ordinator) at Cambridge University Hospitals Trust who were responsible for the delivery of the dance programme.

References

For peer review only - http://bmjopen.bmj.com/site/about/guidelines.xhtml

1. Falvey, J.R., Mangione, K.K., Stevens-Lapsley, J.E. (2015). Rethinking hospital-associated deconditioning: proposed paradigm shift. *Physical therapy*. 1;95(9),1307-15.
2. Aurora, A., Garg, K., Corona, B.T., Walters, T.J. (2014). Physical rehabilitation improves muscle function following volumetric muscle loss injury. *BMC sports science, medicine and rehabilitation*. 6(1), 41.
3. Koenders, N., van Oorsouw, R., Seeger, J.P., Nijhuis–van der Sanden, M.W., van de Glind, I., Hoogeboom, T.J. (2018) "I'm not going to walk, just for the sake of walking…": a qualitative, phenomenological study on physical activity during hospital stay. *Disability and Rehabilitation.* 6,1-8.
4. Oliver, D. (2017). Fighting pyjama paralysis in hospital wards. *BMJ: British Medical Journal* (Online). May 2;357.
5. Clarke, C., Stack, C., & Martin, M. (2018). Lack of meaningful activity on an acute physical hospital wards: Older people's experiences *British Journal of Occupational Therapy* 18(1) 15-23
6. Steele, R., & Linsley, K. (2015). Relieving in-patient boredom in general hospitals: The evidence for intervention and practical ideas. *BJPsych Advances*, 21(1), 63-70. doi:10.1192/apt.bp.113.011908
7. Hanna, J.L. (1983) *The performer-audience connection, emotions and metaphor in dance and society,* Austin: University of Texas
8. Fernández-Argüelles, E.L., Rodriguez-Mansilla. J., Antunez, L.E., Garrido-Ardila, E.M., Muñoz, R.P. (2015). Effects of dancing on the risk of falling related factors of healthy older adults: a systematic review. *Archives of Gerontology and Geriatrics*. 1;60(1), 1-8.

For peer review only - http://bmjopen.bmj.com/site/about/guidelines.xhtml

9. Stacey, G., Stickley, T. (2008). Dancing to keep young@ heart. Mental Health Practice 11(6),34.
10. Ravelin, T., Isola, A., Kylmä, J. (2013). Dance performance as a method of intervention as experienced by older persons with dementia. *International Journal of Older People Nursing.* 8(1),10-8.
11. Vella-Burrows, T., Wilson, L. (2016). Remember to Dance: Evaluating the impact of dance activities for people in different stages of dementia. Canterbury Christ Church University.
12. Vankova, H., Holmerova, I., Machacova, K., Volicer, L., Veleta, P., Celko, A.M. (2014). The effect of dance on depressive symptoms in nursing home residents. *Journal of the American Medical Directors Association.* 15(8), 582-7.
13. da Silva Borges, E.G., de Souza Vale, R.G., Cader, S.A., Leal, S., Miguel, F., Pernambuco, C.S., Dantas, E.H. (2014). Postural balance and falls in elderly nursing home residents enrolled in a ballroom dancing program. *Archives of Gerontology and Geriatrics*. 1;59(2),312-6.
14. Merom, D., Grunseit, A., Eramudugolla, R., Jefferis, B., Mcneil, J., Anstey, K.J (2016). Cognitive benefits of social dancing and walking in old age: The dancing mind randomised controlled trial, *Frontiers in Aging Neuroscience,* doi:10.3389/fnagi.2016.00026.
15. Machacova, K., Vankova, H., Volicer, L., Veleta, P., Holmerova, I. (2017). Dance as prevention of late life functional decline among nursing home residents. *Journal of Applied Gerontology.*36(12),1453-703.
16. Bungay, H, Hughes, S., Jacobs, C., Zheng, J. 2019, Dance for Health: The impact of creative dance sessions on older people in an acute hospital setting *Arts and Health: An*

For peer review only

International Journal for Research Policy and Practice https://doi.org/10.1080/17533015.2020.1725072

17. Bungay et al (in press)
18. Health Research Authority (2016) *Defining Research* available at: http://www.hra-decisiontools.org.uk/research/docs/DefiningResearchTable_Oct2017-1.pdf
19. Clarke, V. & Braun, V. (2014). Thematic analysis. In A. C. Michalos (Ed.), E*ncyclopaedia of Quality of Life and Well-Being Research* (pp. 6626-6628). Springer, Dordrecht, Netherlands: Springer.
20. Keogh, J., W., L., Kilding, A., Pidgeon, P., Ashley, L., Gillis, D., (2012) Effects of different weekly frequencies of dance on older adults functional performance and physical activity patterns. *European Journal of Sports and Exercise Science* 1(1), 14-23
21. Lafrenière, S., Folch, N., Bedard, L., Ducharme, F. (2017). Strategies used by older patients to prevent functional decline during hospitalisation. *Clinical Nursing Research*, 26 (1), 6-26.
22. Baumann, M., Peck, S., Collins, C., Eades, G. (2013). The meaning and value of taking part in a person-centred arts programme to hospitalised stroke patients: Findings from a qualitative study *Disability and Rehabilitation* 35, 244-56.
23. Cortes, O. L., Delgardo, S., Esparza, M. (2019). Systematic review and meta-analysis of experimental studies: In-hospital mobilisation for patients admitted for medical treatment *Journal of Advanced Nursing* 75, 1823-1837.
24. Gregoravic, K. J., Peel, M., Lim, W. K., Hubbard, R. E. (2018). Do health assets have a protective effect for hospitalised frail older patients? *QJM: An International Journal of Medicine,* 111, 11,785-789

For peer review only - http://bmjopen.bmj.com/site/about/guidelines.xhtml

25. MacNaughton, J., White, M., Stacy, R., (2005) Researching the benefits of arts in health. *Health Education* 105(5) 332-339

For peer review only

For peer review only - http://bmjopen.bmj.com/site/about/guidelines.xhtml

Interview Guide Participants

1. How many of the dance (music and movement) sessions that take place on the ward have you joined?
2. Who told you about the group and invited you to join?
3. Do you ever dance when you are at home?
4. Have you had previous experience of dancing? *(Can you tell me what dancing you did and when?)*
5. If you were telling your next door neighbour about the dance (music and movement) sessions on the ward what would you say about them?
6. What are the best bits about the dance (music and movement) sessions?
7. Are there any changes you would like to see? What would make the sessions better?
8. Some people have commented on the following; what do you think about:

   The time/venue/frequency of the sessions?

   How long the session is?

   The music used for the sessions? *(volume? repertoire?)*

   The clarity of explanations? *(How clear is it as to what is happening?)*

9. Some of the participants have mentioned that the sessions affect them in a positive way others find them quite demanding what do you think?

   For example:

   Have you got to know the other patients better?

   Have you got to know the staff better?

   Enjoyed moving to the music?

   Bringing back memories?

   Learning new things?

   Any physical effects (sleep/appetite/fatigue)

10. Is there anything else you would like to say about the dance (music and movement) sessions?

For peer review only

Older people and dance in an acute hospital setting: A qualitative study

Consolidated criteria for reporting qualitative studies (COREQ): 32-item checklist

No. Item	Guide questions/description	Reported on Page #
**Domain 1: Research team and reflexivity**		
Personal Characteristics		
1. Interviewer/facilitator	Which author/s conducted the interview or focus group?	Page 4 both authors conducted interviews
2. Credentials	What were the researcher's credentials? E.g. PhD, MD	Not included on transcript Both authors have PhDs
3. Occupation	What was their occupation at the time of the study?	Not included on transcript<br>Lead Author – Reader in Health and Wellbeing<br>2nd author Research Associate
4. Gender	Was the researcher male or female?	Both female as inferred by names
5. Experience and training	What experience or training did the researcher have?	Not stated inferred by title and qualifications.
Relationship with participants		
6. Relationship established	Was a relationship established prior to study commencement?	Inferred in methods Interviewers had observed dance sessions Page 7
7. Participant knowledge of the interviewer	What did the participants know about the researcher? e.g. personal goals, reasons for doing the research	Potential participants introduced to the study and interviewing team by programme delivery team

For peer review only

8. Interviewer characteristics	What characteristics were reported about the interviewer/facilitator? e.g. Bias, assumptions, reasons and interests in the research topic	Not included in the transcript

For peer review only

**Domain 2: study design**		
Theoretical framework		
9. Methodological orientation and Theory	What methodological orientation was stated to underpin the study? e.g. grounded theory, discourse analysis, ethnography, phenomenology, content analysis	Page 6
Participant selection		
10. Sampling	How were participants selected? e.g. purposive, convenience, consecutive, snowball	Purposive sample Page 6
11. Method of approach	How were participants approached? e.g. face-to-face, telephone, mail, email	Face to face detailed on page 6
12. Sample size	How many participants were in the study?	Page 8
13. Non-participation	How many people refused to participate or dropped out? Reasons?	Not provided – because the initial approached were made by the delivery team the research team do not know if any patients refused to take part
Setting		
14. Setting of data collection	Where was the data collected? e.g. home, clinic, workplace	Page 7 .
15. Presence of non-participants	Was anyone else present besides the participants and researchers?	Inferred on Page 7 stated interview conducted by author 1 or author 2
16. Description of sample	What are the important characteristics of the sample? e.g. demographic data, date	Gender and Age range of participants describe P8
Data collection		

For peer review only

17. Interview guide	Were questions, prompts, guides provided by the authors? Was it pilot tested?	Information provided on page 7<br>Topic guide included as supplementary file
18. Repeat interviews	Were repeat inter views carried out? If yes, how many?	No repeat interviews conducted.
19. Audio/visual recording	Did the research use audio or visual recording to collect the data?	Page 7
20. Field notes	Were field notes made during and/or after the inter view or focus group?	No field notes taken
21. Duration	What was the duration of the inter views or focus group?	Page 7
22. Data saturation	Was data saturation discussed?	This was an exploratory study therefore not looking for data saturation.
23. Transcripts returned	Were transcripts returned to participants for comment and/or correction?	No
**Domain 3: analysis and findings**		
Data analysis		
24. Number of data coders	How many data coders coded the data?	Page 7
25. Description of the coding tree	Did authors provide a description of the coding tree?	No
26. Derivation of themes	Were themes identified in advance or derived from the data?	Page 3 Both inductive and deductive approach
27. Software	What software, if applicable, was used to manage the data?	Page 3
28. Participant checking	Did participants provide feedback on the findings?	No
Reporting		
29. Quotations presented	Were participant quotations presented to illustrate the themes/findings? Was each quotation identified? e.g. participant number	Page 4 to 13 each quotation is identified pseudonyms are used
30. Data and findings consistent	Was there consistency between the data presented and the findings?	Yes, there was.<br>Page 4 to 13

For peer review only

31. Clarity of major themes	Were major themes clearly presented in the findings?	Yes. Three main themes identified
32. Clarity of minor themes	Is there a description of diverse cases or discussion of minor themes?	Minor themes are not presented in this paper

For peer review only

BMJ Open

Older people's perceptions of the impact of Dance for Health sessions in an acute hospital setting: A qualitative study

Journal:	*BMJ Open*
Manuscript ID	bmjopen-2020-044027.R1
Article Type:	Original research
Date Submitted by the Author:	18-Nov-2020
Complete List of Authors:	Bungay, Hilary; Anglia Ruskin University, Allied and Public Health<br>Hughes, Suzanne; Anglia Ruskin University, Allied and Public Health
**Primary Subject Heading**:	Geriatric medicine
Secondary Subject Heading:	Health services research
Keywords:	GERIATRIC MEDICINE, QUALITATIVE RESEARCH, REHABILITATION MEDICINE

SCHOLARONE™
Manuscripts

I, the Submitting Author has the right to grant and does grant on behalf of all authors of the Work (as defined in the below author licence), an exclusive licence and/or a non-exclusive licence for contributions from authors who are: i) UK Crown employees; ii) where BMJ has agreed a CC-BY licence shall apply, and/or iii) in accordance with the terms applicable for US Federal Government officers or employees acting as part of their official duties; on a worldwide, perpetual, irrevocable, royalty-free basis to BMJ Publishing Group Ltd ("BMJ") its licensees and where the relevant Journal is co-owned by BMJ to the co-owners of the Journal, to publish the Work in this journal and any other BMJ products and to exploit all rights, as set out in our licence.

The Submitting Author accepts and understands that any supply made under these terms is made by BMJ to the Submitting Author unless you are acting as an employee on behalf of your employer or a postgraduate student of an affiliated institution which is paying any applicable article publishing charge ("APC") for Open Access articles. Where the Submitting Author wishes to make the Work available on an Open Access basis (and intends to pay the relevant APC), the terms of reuse of such Open Access shall be governed by a Creative Commons licence – details of these licences and which Creative Commons licence will apply to this Work are set out in our licence referred to above.

Other than as permitted in any relevant BMJ Author's Self Archiving Policies, I confirm this Work has not been accepted for publication elsewhere, is not being considered for publication elsewhere and does not duplicate material already published. I confirm all authors consent to publication of this Work and authorise the granting of this licence.

For peer review only

Older people's perceptions of the impact of Dance for Health sessions in an acute hospital setting: A qualitative study

Authors: Hilary Bungay[1]* and Suzanne Hughes[1]

Affiliations: School of Allied and Public Health, Anglia Ruskin University Cambridge

*Corresponding author

Author Email Addresses

- Bungay Hilary: hilary.bungay@anglia.ac.uk
- Hughes Suzanne: suzanne.hughes@anglia.ac.uk

Key Words: Dance, older people, music, movement, hospital, acute care, physical activity, social interaction, emotional support.

Revised manuscript word count: 3998

For peer review only

Original Protocol/topic guide

See attached (supplementary file)

Funding Statement

The Dance for Health Programme was funded by the Dunhill Medical Trust (N187/1116) and included funding for evaluation. The study was commissioned by the Head of Arts via the Addenbrookes Charitable Trust, and the programme delivery team were involved in the study design, but had no input into the data collection, management, analysis or interpretation of the data, review or approvals of the manuscript; or decision to submit the manuscript for publication.

Competing interests

No conflicts of interest declared.

Author Contributions

HB led the study including the design, ethical approval, and observed dance sessions and conducted interviews. SH observed sessions and conducted interviews and contributed to the development of the coding framework. Both HB and SH conducted data analysis and data interpretation. HB wrote the first draft of the manuscript and SH reviewed and suggested improvements to the manuscript draft. Both authors contributed to reviewing and editing the manuscript and approved the final manuscript for submission.

Reporting Checklist

See attached COREQ checklist

For peer review only - http://bmjopen.bmj.com/site/about/guidelines.xhtml

Data Sharing Statement

Data from this study are not being made available for sharing as participants were not asked for specific consent for this as part of the consent process

Patient and Public Involvement

Patients and their relatives or other members of the public were not involved in the design of the study; however, the study was focused on patients and their relatives' perceptions of the programme.

For peer review only

For peer review only - http://bmjopen.bmj.com/site/about/guidelines.xhtml

Abstract

Objectives: To explore the perceptions of hospitalised older people and their relatives of the impact of taking part in group dance sessions in an acute hospital setting.

Design: A qualitative descriptive approach was used.

Setting: An acute hospital Trust in the East of England.

Participants: Purposive sample of dance group participants

Intervention: Weekly music and movement sessions for frail older people on Department of Elderly Medicine Wards and the Stroke Rehabilitation Unit. Sessions take place in the ward and are facilitated by a dance artist supported by ward staff.

Results: Twenty-one semi-structured interviews were conducted with older people and/or their relatives. Thematic analysis of the data identified three overarching themes: (1) Dance as a physical activity, (2) Dance as an opportunity for social interaction and (3) the dance group as a source of emotional support. Sessions were valued as an enjoyable way to undertake physical activity and provided an opportunity for social interaction between patients. This is important as loneliness and boredom are a common occurrence during hospitalisation and are detrimental to overall health and wellbeing. Patients reported an emotional impact though taking part; happiness from engaging with the group, and the release of pent up emotions through the triggering of memories by the music and conversations within the group.

Conclusion: Dance for Health provides a range of physical, social, and emotional benefits for hospitalised older people. Further research is required to investigate the effectiveness of group dance sessions in increasing physical activity on an acute ward, and the potential psychological benefits for hospitalised older people.

For peer review only

Article Summary: Strengths and Limitations of this study

- The first study reporting older people's perspectives of the impact of attending a dance group in an acute hospital setting.
- Only patients who attended the groups were interviewed, the reasons for patients choosing not to join the groups were not captured.
- The study took place in one large teaching hospital in the East of England, this may limit transferability to other institutions and cultures.

For peer review only

For peer review only - http://bmjopen.bmj.com/site/about/guidelines.xhtml

Introduction

When older people are hospitalised, they experience a loss of autonomy, their quality of life can be adversely affected, and they may experience functional decline due to their ability to move around being restricted or limited due to ill health. During hospitalisation older adults may spend 83% of their hospital stay in bed [1] and this lack of mobilisation may result in 'deconditioning syndrome' [2]. The hospital environment discourages physical activity with much of the care taking place at the hospital bedside [3]. In the United Kingdom recognition of this has led to recent initiatives such as "End PJ Paralysis" (where PJ stands for Pyjamas), with patients being encouraged to get out of bed and into their clothes to reduce excessive bed rest [4]. The restricted opportunity for movement can also mean that there is little opportunity for engagement in social interaction. This can lead to boredom, a common occurrence in hospitalised older adults, resulting in low mood [5] and cognitive decline [6].

Dance is a physical activity which not only employs motor skills, but also engages cognitive functions, including perception, emotions, and memory [7]. Research on dance with older people, has been conducted in a range of settings and across the globe [8-14]. But there are a limited number of studies that include frail or functionally impaired adults [15]. Furthermore, not all studies have identified positive outcomes. A Randomised Controlled Trial looking at social dancing and the incidence of falls in older people found that dance participants experienced a higher rate of falls than the control group [14]. There are also methodological weaknesses in some of the existing research with small sample sizes, and heterogeneity of tools and scales used in quantitative studies and a lack of transparency in the reporting of qualitative data analysis [8].

A large hospital Trust in the East of England introduced a weekly *Dance for Health* programme. The underpinning philosophy for *Dance for Health* is that it is about dance and

For peer review only - http://bmjopen.bmj.com/site/about/guidelines.xhtml

enabling people to be creative and expressive rather than being just an exercise programme or a therapeutic intervention. The sessions are facilitated by a dance artist and take place weekly on wards in the Department of Elderly Medicine and the Stroke and Rehabilitation Unit. The sessions are open to all inpatients on the wards where the programme takes place including people with Parkinson's Disease, delirium, unstable diabetes, stroke, falls, and dementia. A description of the format of the sessions is presented in the supplementary file 1.

Method

Research Design

A qualitative exploratory descriptive approach within an interpretive framework using semi-structured interviews was used to explore patients' perspectives and experiences of participating in *Dance for Health.* The study data presented here formed part of a larger service evaluation which adopted a mixed methods approach, including structured observation and interviews with health care professionals [16, 17].

A pragmatic approach to sample size and interview method was adopted, taking account of the range of data collection methods in the study, the potential diversity of participants, demands on the participants and the local context [18]. Purposive sampling was used to ensure only patients and/or relatives who had attended the dance sessions were included in the study. Hospital staff identified potential participants and introduced them to the evaluation team. Inclusion criteria included one or more attendance at a dance session, and the ability to provide consent. Patients or relatives unable to consent to interview or who had not attended a session were excluded from the research. Participants received a participant information sheet and provided written consent to be interviewed. HB or SH observed the dance sessions as part of the wider study and so were recognised by the participants. They had no personal or professional contact with the patients beyond those sessions. The research team were not

For peer review only - http://bmjopen.bmj.com/site/about/guidelines.xhtml

informed if potential participants had refused to be interviewed. As an evaluation involving an intervention in current use meeting the Health Research Authority criteria of an evaluation [19], the project was registered with the Safety and Quality Support Department at the Cambridge University Hospitals Trust (PRN:6594). It was also approved by the ARU University Faculty Research Ethics Panel (FREP).

Data Collection

To answer the objectives of the study funders and dance programme delivery team, participants were asked about their likes/dislikes about the sessions and the effects they felt taking part had on their overall wellbeing and physical health (see supplemental file 2 interview guide). The interviews were semi-structured and conducted face to face and the length ranged from 5-33 minutes. Most interviews took place following dance sessions, except four which took place the following day. Interviews were conducted in the day room where the activity had taken place or in the ward bay. The interviews and data analysis were undertaken by HB and SH. HB (she/her) is an Associate Professor in Health and Wellbeing and has undertaken qualitative research with patients, service users and staff across a range of health and community settings over the last 20 years. SH (she/her) is an experienced post-doctoral research associate who has untaken qualitative research with patients, service users and informal carers. The interviews were audio-recorded and transcribed verbatim for analysis. Transcripts were anonymised using pseudonyms and checked for accuracy against the original recording.

Data Analysis

The data analysis process followed an interpretive approach, and the data was explored to ensure the analysis reflected the accounts of the participants and responded to new themes as identified. Thematic analysis followed Clarke and Braun's six stages of analysis and was conducted systematically to ensure rigour and trustworthiness [20]. To increase dependability

For peer review only - http://bmjopen.bmj.com/site/about/guidelines.xhtml

and credibility the first three interviews were listened to by both authors for consistency before further interviews were undertaken. To further ensure dependability transcripts were checked by HB for accuracy with the audio recordings. This also familiarised the authors with the data. To ensure trustworthiness of analysis both authors independently read each interview transcript and made notes of potential codes, and through discussion a coding framework was created. The framework was applied across the data set with detailed coding by HB and the coded data was then searched for patterns to develop sub-themes which were then clustered and organised into the final themes by HB and SH. The table in Supplementary File 2 provides an example of the coding process, and the development of a sub-theme and theme.

Results

Twenty-one patients and/or relatives were interviewed by either HB or SH, who had also attended sessions and undertaken structured observations. Two of those interviewed were men, and the age of those interviewed ranged from 60s to 92. Participants were not asked about the reasons for their admission to hospital or their ages, but some volunteered their age during the interview and the ages of other participants were estimated (by decade) by the team during the interview interaction.

From the analysis, three main themes related to the perceived impact of the sessions were identified: Dance as a physical activity, Dance as an opportunity for social interaction and the dance group as a source of emotional support (see figure in Supplementary File 2). Where relevant the reported findings are supported with observations from the sessions. The names used throughout are pseudonyms to protect the identity of the participants.

Dance as a physical activity

Patients participating in the sessions were elderly and mostly frail, with restricted mobility due to their physical health. The majority remained seated throughout the dance sessions, moving

For peer review only - http://bmjopen.bmj.com/site/about/guidelines.xhtml

their bodies to varying degrees to the music. Nearly two thirds (13) of those interviewed, when asked to describe what they liked about the sessions, referred to the physical impact and the opportunity for activity the sessions provided. The dance sessions were seen to be beneficial in terms of loosening up stiff muscles and joints, with recognition that being in bed for long periods of time leads to the body becoming stiff and resistant to movement.

"Yes, everything. Yes, because it helps you to move your limbs and get you going. As I said, I've got no use- my hands lock, and you drop things sometimes. The exercise helps your hands, everything….. Yes, get your body going". (Ellen)

"It just helps everything. Helps the muscles, and the mind and everything….it just relaxes you" (Angela)

The music which accompanies the sessions encourages movement, and participants would sway in time to the music and mirror the movements of the dance artist and others in the group. Many of the participants would improvise their own moves with others copying these actions. Even patients who were very frail and unable to make extensive movements would adjust their posture and would sit up being noticeably more upright in their chairs. For some, upbeat music encouraged rhythmic movement, whilst others responded to slower tracks by stretching their limbs.

"You could think with the music and you could develop your own system of stretches, it sort of guided you into the movements you see. Yes, the music guided you into different things to think of the areas that needed stretching, you see, the bits that hurt. Some of the music gave you the impetus to move and stretch……..You are doing like you are in the water, like you are swimming, that you're in freestyle". (Carol).

Whilst some attributed their movements in the sessions to the music that was played, it was also seeing others in the group move which inspired them to try and move more.

For peer review only - http://bmjopen.bmj.com/site/about/guidelines.xhtml

"Yes, power in it, in the music. To be honest with you, it is the music that makes you want to dance, you know?" (Maureen)

"It's uplifting, and it's just rewarding, and movement was great because I fell on my right side of my body. So, just moving my arms slowly was fantastic, but I met the other patients there, not all from (ward 1), some from (ward 2). It was lovely, and I thought, you know, "I just feel horrible, and I feel I'm the only sad person in here," and you look at other people, and some people are worse. I'm really grateful. Not grateful that I saw them, but these other people are struggling as well… Looking at other people and thinking, "Well, you know what? I can do this and I can do that." (Alex)

"Wanting to do something. Even just standing up in the chair, suddenly, was a massive thing for people, but they wanted to do it, because they felt motivated, inspired, feeling a bit alive, by the whole situation". (Relative 2)

This relative also went on to suggest that the sessions were like a form of physiotherapy without participants realising that they were engaging in physiotherapy.

It was interesting that those interviewed referred to how they felt other patients benefited from taking part in the sessions. This mostly related to enjoyment, but also the benefits of the physical movement.

"Oh lots of ways, but it helps them to get moving and moving their limbs, and I've seen (name) lifting her legs up and things like that" (Ellen)

"Everyone was able to do the movements that you gave, and going from the sublime to a bit more jazzy it was beautiful". (Mary)

Three of those interviewed referred specifically to exercise, including Collette who called the movements' 'exercises'.

For peer review only - http://bmjopen.bmj.com/site/about/guidelines.xhtml

"I think, everybody joined in and were very happy, and they did their exercises, which is lovely. It was very, very nice".

Dance as an opportunity for social interaction

While the beneficial physical impact of the sessions was acknowledged by participants, the sessions were also valued for the opportunity for the social interactions the sessions provided. The dance activity takes place in a circle formation so people are sitting close to each other and can see others in the group. Often a sense of fun was apparent, as many of the interactions generated laughter within the group. Such group interactions had a positive effect on those interviewed…

"I thought it was very therapeutic…. Something up here made you feel you were with it and you felt involved. That's how I felt. I didn't feel lonely or shut out. I felt it was company" (June)

"We were laughing and talking as well, and I think that helped too. It generated a community feeling, do you know what I mean? (Margaret)

Others remarked that it had given them an opportunity to get to know other people on the ward and the two men particularly valued this.

"I found the session today, which I was a bit doubtful about to start with, very interesting. Because to start with, I learnt the names of patients who were in the same ward as me but who you don't see when you're lying in bed all day….I learnt their names and I found the music had an enlivening effect on everybody…".(David)

"Even those who were perhaps more disabled than others. So I thought it was a very good session. I thought we bonded together….( ) Well, you're lying in bed and when you can't get up and walk around, you can't talk to people." (Lionel)

For peer review only - http://bmjopen.bmj.com/site/about/guidelines.xhtml

For peer review only

The impact of the session extended beyond the allotted time for the session. In one ward, three women who were taking part in the session just before lunch struck up a conversation, and decided to stay in the day room following the session to have their lunch together. One of the relatives interviewed also described how, following a session, people in the ward bay who had joined the session were talking about it during the rest of the day. She also remarked how they found common ground because of the session and the mood was buoyant because of joining in. Another relative who was interviewed with her mother also commented on how it was good that the patients could get together in a fun activity.

"I thought it was brilliant. And I think it's so good to get the people together as well because they interact with each other and they do things together and it's fun. I thought it was brilliant". (Relative 1)

This same relative also commented on how she liked the fact that she was able to do the session with her mother, and how it gave them something to do together, which was increasingly unusual as her mother aged.

The dance artist would ask people if they would like to hear a piece of music and this would often trigger conversations as people requested certain tracks. In addition, when people recognised certain tunes this could evoke memories that initiate further conversations and laughter in the group. Through these conversations people got to know each better and, in some cases,, they found that they had previously been at the same school or lived in the same road many years ago. As well as providing a sense of community for individuals who experienced loneliness at home, the sessions gave people the opportunity to make new friends

"I enjoyed being with the people, different people" (Margaret)

"It's fantastic being with other people and just chat with other people" (Alex)

For peer review only - http://bmjopen.bmj.com/site/about/guidelines.xhtml

"Because I am registered disabled, so don't go a lot. I used to be sociable, and I have lost all my friends now, so this is really nice" (Polly)

The dance groups as a source of emotional support

The sessions were often happy and relaxed with conversations and different songs generating amusement and laughter. However, for some participants the music triggered sadness and sometimes tears, although for others the tears were brought on through happiness. One such case was observed when a patient said at the end of a song "*it brought tears to my eyes.. I love togetherness, sharing happiness"* in another example when dancing to 'Love me Tender' one patient cried and said *"but I enjoyed it … it really touched me".* One of the men who was interviewed reflected on how the songs reminded him of his youth, making him feel sad.

"I felt a bit tearful when we played songs that took me back to my youth, really". (Lionel)

Although some patients did become sad when certain songs were played, this was not necessarily a negative impact. For example, Maud said that since she had been in the hospital she had felt that she had had a lump in her chest because she was bottling her emotions and concerns about what was wrong with her. She went on to explain that she had cried in the music and dance session, experiencing a sense of release and feeling that it was safe to cry in front of others who were going through the same thing as herself.

"It was very uplifting, and you know. Tearful for me... Everything bottles up, I think. Coming in hospital, and it sort of brings it out. And its cleansing. I feel better for it….because I kind of had a lump here, that it was bottling up…I didn't know what the outcome of that was going to be. So, I just found it very, as I said, uplifting and healing. I don't know. I think lots of stuff goes on in your head when you come into hospital. When you don't know really what's wrong. ….. but it's a worrying time for the family. Yes, bottled up, and you know. But being able to talk about it. And cry, you know, even cry in silence. It's just cleansing. Being here, yes. Among

For peer review only - http://bmjopen.bmj.com/site/about/guidelines.xhtml

other people who are going through the same, or more or less the same things, you know". (Maud)

If participants were upset the rest of the group were always very supportive and offered words of encouragement, this included touching the person's arm or taking hold of a hand. Such actions are a form of emotional support that is provided by a being part of group and can help people to cope with stressful situations.

Most participants however, appeared to really enjoy the sessions, and spoke about how much they had enjoyed the dance and found it uplifting,

"and I really enjoyed that. It really lifted up my spirits. Honestly, I love it. I've been feeling so low, and it just helped me build up, and I feel so positive now". (Alex)

"It made me feel so wonderful afterwards. I felt like I was walking on air". (Polly)

"It was very enjoyable. I found it relaxing and reviving at the same time, if you know what I mean. And thinking and coordinating and everything else which is something you don't practice". (Dora)

"...It was a real enjoyable session. I really enjoyed it. ...Bearing in mind that I've been stuck in a room in a hospital for about four or five months. This was an outlet". (David)

"I thought it was brilliant. [....] Yes, it really gave me a lift", (Gertie)

Again, participants also talked about the impact on others of taking part and how they had observed people coming to the sessions 'grouchy' and 'grumpy', yet through taking part their demeanour changed significantly and people appeared more cheerful and happier.

"and all the other patients were lively and bright too, what they were when they came in. They were all flat and miserable, and they livened up. They were totally different. Yes. They looked totally different when they left". (Mary)

For peer review only - http://bmjopen.bmj.com/site/about/guidelines.xhtml

Discussion

In this study, the dance sessions were perceived to be an activity which provided participants with an opportunity to increase their levels of physical activity. Patients enjoyed the opportunity to interact with others on the ward, and despite some sadness, the majority enjoyed the sessions and experienced a positive affect through taking part.

The perceptions of those interviewed were that the dance sessions were beneficial to their health and wellbeing. A major strength of this study is that it presents the findings from an innovative programme of music and movement for older people in an acute hospital setting. Semi-structured interviews with patients and relatives provide insight into the perceived benefits of the programme, and a rationale for exploring how such programmes could be introduced to increase levels of physical activity and to help alleviate patient boredom on acute hospital wards.

As with all research there are limitations to this study. The interviews were semi-structured and relatively short, not only because they were designed to answer specific questions for the funder, but also because participants were frail and easily fatigued. Unstructured interviews may have enabled a more in-depth understanding of the patients experiences and how these related to their perceptions about the programme. Similarly, data was not collected regarding individuals' functional status or on-going treatments and this may have provided a useful dimension to the research. Furthermore, only patients who participated in groups were interviewed and their responses were all very positive. It would have been interesting to interview those who chose not to attend the sessions and to explore their reasons, particularly as more women than men attended the sessions each week.

The patients participating in the Dance for Health groups were often very elderly (85+) and frail, and had been hospitalised for a number of weeks, and so remained seated for the activity.

For peer review only - http://bmjopen.bmj.com/site/about/guidelines.xhtml

We could find no evidence in the literature on the effectiveness of seated dance as a physical activity for older people, although a systematic review found that dance can have multiple physical benefits for older people, including aerobic power, muscle endurance, strength and flexibility of lower body and balance [21]. Our participants expressed understanding of the need to try and maintain their physical abilities. This resonates with the work of Lafrenière et al., who found that older hospitalised adults were cognisant of the risks of functional decline utilising various strategies to maintain both physical abilities and good spirits [22]. Experiencing things that are familiar and 'normal', such as dance and listening to music, when in an uncertain and stressful environment can also help people to maintain a sense of self. Furthermore, pleasure and enjoyment, a sense of connection, are linked to mental wellbeing [23], and social engagement and improving psychological health has been found to lead to positive health outcomes [24].

It has been recommended that purposeful meaningful activities should be included in multi-component interventions designed to increase physical activity of patients during a hospital stay [3]. As outlined previously, hospitalised older people can have low levels of activity, which results in deconditioning, and can lead to longer hospital stays, and possible further loss of physical function affecting recovery post-discharge [25]. It is therefore imperative that care providers consider alternative forms of exercise such as dance to enable and encourage hospitalised older people to engage in physical activity. A group activity such as dance, which is seen as fun rather than therapy could be an attractive option for patients, particularly as the arts tend to be associated with normative pleasurable experiences making them more attractive, accessible and acceptable than conventional care [26].

Conclusion

For peer review only - http://bmjopen.bmj.com/site/about/guidelines.xhtml

This paper presents the findings from a study of an innovative programme of dance activities introduced onto Elderly Medicine wards and Stroke and Rehabilitation Unit in an acute hospital Trust. As discussed in the introduction, there exists research which demonstrates the impact of dance on the health and wellbeing of older people in the community and in residential care. The study focussed on the experiences of hospitalised older people and their relatives taking part in the dance sessions. It was not possible to conduct a controlled study to look at the impact on the number of falls, whether physical activity increased, or if the rate of rehabilitation improved because of taking part. However, what the study demonstrated is the value placed on the activity by the participants in terms of positive affect and the social opportunity the sessions afforded. In addition, and of significance, is also how the patients recognised the importance of maintaining physical function and how the dance activity encouraged them to move. Further research exploring the use of dance with hospitalised older people would be valuable to investigate whether overall physical activity levels do increase as a result of the dance activity, with measurement of both physical functioning, and the psychological wellbeing of participants undertaken.

Acknowledgements

We would like to thank the patients who were interviewed for this study, and Damian Hebron (Head of Arts), Filipa Pereira-Stubbs (Dance artist), and Deborah Quartermaine (Project Co-ordinator) at Cambridge University Hospitals Trust who were responsible for the delivery of the dance programme.

References

1. Falvey JR, Mangione KK, Stevens-Lapsley JE. Rethinking hospital-associated deconditioning: proposed paradigm shift. *Physical Therapy* 2015;95(9):1307-15.

For peer review only - http://bmjopen.bmj.com/site/about/guidelines.xhtml

2. Aurora A, Garg K, Corona BT, Walters TJ. Physical rehabilitation improves muscle function following volumetric muscle loss injury. *BMC Sports Science, Medicine and Rehabilitation* 2014; 6(1): 41.
3. Koenders N, van Oorsouw R, Seeger JP, Nijhuis–van der Sanden MW, van de Glind I, Hoogeboom TJ. "I'm not going to walk, just for the sake of walking…": a qualitative, phenomenological study on physical activity during hospital stay. *Disability and Rehabilitation* 2018; 6:1-8.
4. Oliver D. Fighting pyjama paralysis in hospital wards. *BMJ: British Medical Journal* (Online) 2017;357.
5. Clarke C, Stack C, Martin M. Lack of meaningful activity on an acute physical hospital wards: Older people's experiences, *British Journal of Occupational Therapy* 2018; 18(1):15-23.
6. Steele R, Linsley K. Relieving in-patient boredom in general hospitals: The evidence for intervention and practical ideas. *BJPsych Advances* 2015; 21(1):63-70. doi:10.1192/apt.bp.113.011908
7. Hanna JL. The performer-audience connection: Emotion to metaphor in dance and society. Austin: University of Texas Press; 1983 Nov 1.
8. Fernández-Argüelles EL, Rodriguez-Mansilla J, Antunez LE, Garrido-Ardila EM, Muñoz RP. Effects of dancing on the risk of falling related factors of healthy older adults: a systematic review. *Archives of Gerontology and Geriatrics* 2015;60(1):1-8.
9. Stacey G, Stickley T. Dancing to keep young@ heart. *Mental Health Practice* 2008;11(6):34.
10. Ravelin T, Isola A, Kylmä J. Dance performance as a method of intervention as experienced by older persons with dementia. *International Journal of Older People Nursing* 2013; 8(1): 8-10.

For peer review only - http://bmjopen.bmj.com/site/about/guidelines.xhtml

11. Vella-Burrows T, Wilson L. *Remember to Dance: Evaluating the impact of dance activities for people in different stages of dementia*. Canterbury Christ Church University, 2016.

12. Vankova H, Holmerova I, Machacova K, Volicer L, Veleta P, Celko AM. The effect of dance on depressive symptoms in nursing home residents. *Journal of the American Medical Directors Association* 2014; 15(8): 582-7.

13. da Silva Borges EG, de Souza Vale RG, Cader SA, Leal S,Miguel F, Pernambuco CS, Dantas EH. Postural balance and falls in elderly nursing home residents enrolled in a ballroom dancing program. *Archives of Gerontology and Geriatrics* 2014; 59(2):312-6.

14. Merom D, Mathieu E, Cerin E, Morton RL, Simpson JM, Rissel C, Anstey K J, Sherrington C, Lord S R ,Cumming R G. Social Dancing and Incidence of Falls in Older Adults: A Cluster Randomised Controlled Trial. *PLoS Med.* 2016;13(8):e1002112. doi:10.1371/journal.pmed.1002112

15. Machacova K, Vankova H,Volicer L, Veleta P,Holmerova I. Dance as prevention of late life functional decline among nursing home residents. *Journal of Applied Gerontology* 2017; 6(12):1453-703.

16. Bungay H, Hughes S, Jacobs C, Zheng J. Dance for Health: The impact of creative dance sessions on older people in an acute hospital setting *Arts and Health: An International Journal for Research Policy and Practice* 2019.https://doi.org/10.1080/17533015.2020.1725072

17. Bungay H, Jacobs C. Dance for Health: The perceptions of healthcare professionals of the impact of music and movement sessions for older people in an acute hospital setting. *International Journal of Older People Nursing* 2020. http://dx.doi.org/10.1111/opn.12342

For peer review only - http://bmjopen.bmj.com/site/about/guidelines.xhtml

18. Braun V, Clarke V. To saturate or not to saturate? Questioning data saturation as a useful concept for thematic analysis and sample-size rationales. *Qualitative Research in Sport, Exercise and Health* 2019.doi: 10.1080/2159676X.2019.1704846

19. Health Research Authority *Defining Research* 2016 available at: http://www.hra-decisiontools.org.uk/research/docs/DefiningResearchTable_Oct2017-1.pdf

20. Clarke V, Braun V. Thematic analysis in: Michalos AC (Ed.). Encyclopaedia of Quality of Life and Well-Being Research 2014;6626-6628. Springer, Dordrecht, Netherlands: Springer.

21. Keogh JWL, Kilding A, Pidgeon P, Ashley L, Gillis D. Effects of different weekly frequencies of dance on older adults functional performance and physical activity patterns. *European Journal of Sports and Exercise Science* 2012; 1(1):14-23.

22. Lafrenière S, Folch N, Bedard L, Ducharme F. Strategies used by older patients to prevent functional decline during hospitalisation. *Clinical Nursing Research* 2017;26 (1): 6-26.

23. Baumann M, Peck S, Collins C, Eades G. The meaning and value of taking part in a person-centred arts programme to hospitalised stroke patients: Findings from a qualitative study *Disability and Rehabilitation* 2013;35: 244-56.

24. Cortes OL, Delgardo S, Esparza M. Systematic review and meta-analysis of experimental studies: In-hospital mobilisation for patients admitted for medical treatment. *Journal of Advanced Nursing* 2019;75:1823-1837.

25. Gregoravic KJ, Peel M, Lim WK, Hubbard RE. Do health assets have a protective effect for hospitalised frail older patients? *QJM: An International Journal of Medicine* 2018;111(11):785-789.

26. MacNaughton J, White M, Stacy R. Researching the benefits of arts in health. *Health Education* 2005;105(5):332-339.

For peer review only - http://bmjopen.bmj.com/site/about/guidelines.xhtml

For peer review only

For peer review only - http://bmjopen.bmj.com/site/about/guidelines.xhtml

Interview Guide Participants

1. How many of the dance (music and movement) sessions that take place on the ward have you joined?
2. Who told you about the group and invited you to join?
3. Do you ever dance when you are at home?
4. Have you had previous experience of dancing? *(Can you tell me what dancing you did and when?)*
5. If you were telling your next door neighbour about the dance (music and movement) sessions on the ward what would you say about them?
6. What are the best bits about the dance (music and movement) sessions?
7. Are there any changes you would like to see? What would make the sessions better?
8. Some people have commented on the following; what do you think about:

   The time/venue/frequency of the sessions?

   How long the session is?

   The music used for the sessions? *(volume? repertoire?)*

   The clarity of explanations? *(How clear is it as to what is happening?)*

9. Some of the participants have mentioned that the sessions affect them in a positive way others find them quite demanding what do you think?

   For example:

   Have you got to know the other patients better?

   Have you got to know the staff better?

   Enjoyed moving to the music?

   Bringing back memories?

   Learning new things?

   Any physical effects (sleep/appetite/fatigue)

10. Is there anything else you would like to say about the dance (music and movement) sessions?

For peer review only

Supplementary File

Description of the Dance for Health Programme

The Dance for Health for older people programme takes place on wards in the Department of Elderly Medicine ward, the Diabetes and Endocrine Medicine ward, and the Stroke and Rehabilitation Unit in a large acute hospital Trust. The dance sessions are weekly and scheduled for an hour on each ward and usually take place in the ward day rooms. The number attending the sessions ranges between 4-12 patients and is dependent on the available space and staff support. The sessions are introduced to patients by the ward staff as music and movement. There is no exercise regime imposed and no choreography. Patients are invited to participate in the activity to the degree that they feel comfortable and choose whether to attend the sessions or not.

The dance sessions aim to improve the hospital experience of older people and to enhance their wellbeing and physical health. They are delivered by a Dance Artist who is a trained Dance Movement Therapist, but the sessions are not offered as a psychotherapeutic intervention. The Project Coordinator, who is also the Falls Prevention Co-ordinator for the Trust, supports the sessions through both liaising with wards where the sessions take place and by helping wards to identify patients who may benefit from taking part in the sessions. The sessions are open to all inpatients on the wards where the programme takes place, including people with Parkinson's disease, delirium, unstable diabetes, stroke, falls and dementia. The Dance Artist and Project Coordinator provided information and training for ward staff to raise awareness of the programme and engage staff in the sessions.

The dance activity takes place in a circle formation so participants are close to the person next to them and can see others in group. The sessions always start with the Dance Artist introducing the session and stating the day of the week and the date, followed by the location of the activity. She then asks all those present to introduce themselves in turn. The first music track played is an instrumental piece and the participants are encouraged to start some gentle movements to warm up their hands, arms, and shoulders. During the following piece of music, lower body movement is encouraged. Participants are then invited to choose a music track; this means that some of the music is familiar to those taking part. The process of choosing music also helps to stimulate conversation between the participants. The middle of the session is livelier, using music with a more upbeat tempo. This encourages more movement and participants may also

For peer review only

join in singing to some of the tracks. Towards the end of the session, slower quieter music is used during the cool down phase and to help relax the participants. During this relaxation time, patients are given a gentle massage to their neck and shoulders by the staff present, and participants are invited to close their eyes and listen to the quiet, gentle, instrumental music played at this time.

Table 1: Example of the coding process and theme development

Data Extract	Code	Sub-Theme	Theme
"You could think with the music and you could develop your own system of stretches, it sort of guided you into the movements you see. Yes, the music guided you into different things to think of the areas that needed stretching, you see, the bits that hurt. Some of the music gave you the impetus to move and stretch……You are doing like you are in the water, like you are swimming, that you're in freestyle".	Moving and stretching<br>Inspired to move	Encourages Movement	Physical Impact

Figure 1: Thematic Map

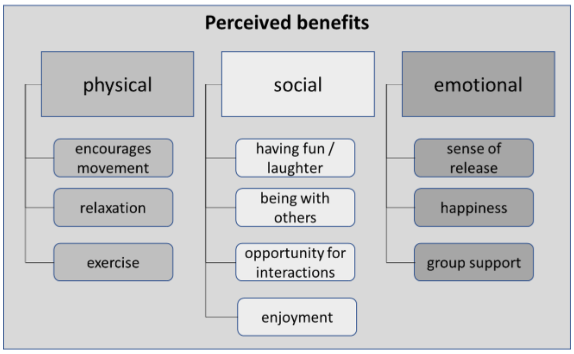

For peer review only

Older people's perceptions of the impact of Dance for Health sessions in an acute hospital setting: A qualitative study

Consolidated criteria for reporting qualitative studies (COREQ): 32-item checklist

No. Item	Guide questions/description	Reported on Page #
**Domain 1: Research team and reflexivity**		
Personal Characteristics		
1. Interviewer/facilitator	Which author/s conducted the interview or focus group?	Both authors conducted interviews
2. Credentials	What were the researcher's credentials? E.g. PhD, MD	Both authors have PhDs, Masters and professional qualifications
3. Occupation	What was their occupation at the time of the study?	Lead Author – Associate Professor 2nd author Research Associate
4. Gender	Was the researcher male or female?	Preferred pronouns she/her
5. Experience and training	What experience or training did the researcher have?	Both experienced in conducting qualitative research and interviews.
Relationship with participants		
6. Relationship established	Was a relationship established prior to study commencement?	Interviewers had observed the dance sessions which the participants attended
7. Participant knowledge of the interviewer	What did the participants know about the researcher? e.g. personal goals, reasons for doing the research	Potential participants introduced to the study and research team at the beginning of each dance session
8. Interviewer characteristics	What characteristics were reported about the interviewer/facilitator? e.g. Bias,	Occupation and experience included

For peer review only

	assumptions, reasons and interests in the research topic	

For peer review only

**Domain 2: study design**		
Theoretical framework		
9. Methodological orientation and Theory	What methodological orientation was stated to underpin the study? e.g. grounded theory, discourse analysis, ethnography, phenomenology, content analysis	An exploratory descriptive study
Participant selection		
10. Sampling	How were participants selected? e.g. purposive, convenience, consecutive, snowball	Purposive sampling was used
11. Method of approach	How were participants approached? e.g. face-to-face, telephone, mail, email	Face to face interviews and recruitment
12. Sample size	How many participants were in the study?	21
13. Non-participation	How many people refused to participate or dropped out? Reasons?	Because the initial approached were made by the ward staff and delivery team the research team do not know if any patients refused to take part
Setting		
14. Setting of data collection	Where was the data collected? e.g. home, clinic, workplace	In a hospital setting .
15. Presence of non-participants	Was anyone else present besides the participants and researchers?	Only the participants and researchers were present
16. Description of sample	What are the important characteristics of the sample? e.g. demographic data, date	The gender and age range of participants are outlined
Data collection		

For peer review only

17. Interview guide	Were questions, prompts, guides provided by the authors? Was it pilot tested?	A Topic guide was used and is included as supplementary file
18. Repeat interviews	Were repeat inter views carried out? If yes, how many?	No repeat interviews were conducted.
19. Audio/visual recording	Did the research use audio or visual recording to collect the data?	The interviews were audio recorded
20. Field notes	Were field notes made during and/or after the inter view or focus group?	No field notes taken
21. Duration	What was the duration of the interviews or focus group?	Interviews ranged form 5-33 minutes
22. Data saturation	Was data saturation discussed?	This was an exploratory study therefore not looking for data saturation.
23. Transcripts returned	Were transcripts returned to participants for comment and/or correction?	No – it was not possible to return the transcripts to the participants
**Domain 3: analysis and findings**		
Data analysis		
24. Number of data coders	How many data coders coded the data?	Two data coders coded the data
25. Description of the coding tree	Did authors provide a description of the coding tree?	A thematic map is included
26. Derivation of themes	Were themes identified in advance or derived from the data?	Page 3 Both inductive deductive approaches were used
27. Software	What software, if applicable, was used to manage the data?	Software was not used to manage the data
28. Participant checking	Did participants provide feedback on the findings?	No this was not possible
Reporting		

For peer review only

29. Quotations presented	Were participant quotations presented to illustrate the themes/findings? Was each quotation identified? e.g. participant number	Quotationa are presented to illustrate themes and pseudonyms are used to protect identity of participants
30. Data and findings consistent	Was there consistency between the data presented and the findings?	Yes, there is congruence between the data presented and the findings
31. Clarity of major themes	Were major themes clearly presented in the findings?	Three main themes were identified and are presented in the findings
32. Clarity of minor themes	Is there a description of diverse cases or discussion of minor themes?	Minor themes are not presented in this paper

For peer review only
